# Renal involvement in autoimmune connective tissue diseases

**DOI:** 10.1186/1741-7015-11-95

**Published:** 2013-04-04

**Authors:** Andreas Kronbichler, Gert Mayer

**Affiliations:** 1 Department of Internal Medicine IV, Nephrology and Hypertension, Medical University Innsbruck, Anichstraße 35, Innsbruck, 6020, Austria

**Keywords:** Renal involvement, Connective tissue diseases, Sjögren syndrome, Scleroderma renal crisis, Dermatomyositis/polymyositis, Systemic lupus erythematosus, Antiphospholipid syndrome, Rheumatoid arthritis

## Abstract

Connective tissue diseases (CTDs) are a heterogeneous group of disorders that share certain clinical presentations and a disturbed immunoregulation, leading to autoantibody production. Subclinical or overt renal manifestations are frequently observed and complicate the clinical course of CTDs. Alterations of kidney function in Sjögren syndrome, systemic scleroderma (SSc), auto-immune myopathies (dermatomyositis and polymyositis), systemic lupus erythematosus (SLE), antiphospholipid syndrome nephropathy (APSN) as well as rheumatoid arthritis (RA) are frequently present and physicians should be aware of that.

In SLE, renal prognosis significantly improved based on specific classification and treatment strategies adjusted to kidney biopsy findings. Patients with scleroderma renal crisis (SRC), which is usually characterized by severe hypertension, progressive decline of renal function and thrombotic microangiopathy, show a significant benefit of early angiotensin-converting-enzyme (ACE) inhibitor use in particular and strict blood pressure control in general. Treatment of the underlying autoimmune disorder or discontinuation of specific therapeutic agents improves kidney function in most patients with Sjögren syndrome, auto-immune myopathies, APSN and RA.

In this review we focus on impairment of renal function in relation to underlying disease or adverse drug effects and implications on treatment decisions.

## Background

Impairment of renal function is present to some extent in many connective tissue diseases (CTDs) with variable occurrence in Sjögren syndrome [1,2], roughly 5% in systemic scleroderma (SSc) [3], rarely in inflammatory auto-immune myopathies, a prevalence of approximately 50% in systemic lupus erythematosus (SLE) [4], and rare occurrence in antiphospholipid syndrome [5] and rheumatoid arthritis (RA). Apart from that, kidney involvement can be of significant prognostic value and often entails specific therapeutic implications.

Lymphocytic infiltration, leading to acute or chronic tubulointerstitial nephritis, is the predominant renal pathology in Sjögren syndrome [2,6,7]. Scleroderma renal crisis (SRC) is a severe, potentially life-threatening complication in scleroderma and is, in most cases, accompanied by malignant hypertension, overexpression of pro-inflammatory cytokines and rapid decline of renal function [8-10]. In rare cases patients present with normotensive SRC, which is associated with a poorer prognosis and a prompter need for dialysis [11-13]. Early commencement of angiotensin-converting-enzyme (ACE)-inhibitors and other antihypertensive drugs is mandatory in the management of SRC. Rhabdomyolysis with acute tubular necrosis or glomerular disorders, including minimal change disease, membranous nephropathy, IgA nephropathy or diffuse proliferative glomerulonephritis, has been reported in patients with auto-immune myopathies [14,15].

Lupus nephritis is one of the most severe organ manifestations of the disease and, depending on biopsy findings, needs aggressive immunosuppressive therapy. The histopathologic classification of lupus nephritis guides therapeutic interventions with the aim to reduce proteinuria and preserve kidney function. Renal manifestation in primary and secondary antiphospholipid syndrome (APS) is a well-described complication, frequently leading to arterial hypertension and occasionally impairment of renal function [5,16]. Patients with RA are at an increased risk of developing secondary amyloidosis due to long-lasting chronic inflammation as well as mesangial glomerulonephritis and membranous nephropathy related to specific drugs [17]. Table 1 summarizes specific kidney biopsy findings in the context of CTDs.

**Table 1:** Overview of kidney biopsy findings in patients with connective tissue diseases

**Renal biopsy findings**	**Connective tissue disease**
**Tubulointerstitial nephritis (TIN)**	Sjögren syndrome [2,6,7], Rheumatoid arthritis [17]
**Mesangial proliferative glomerulonephritis/IgA nephropathy**	Sjögren syndrome [2], Polymyositis [46,50,51], Dermatomyositis [58], Systemic lupus erythematosus [80], Rheumatoid arthritis [17,138]
**Focal segmental glomerulosclerosis (FSGS)**	Sjögren syndrome [6], Systemic lupus erythematosus [81], Antiphospholipid syndrome [124], Rheumatoid arthritis [139]
**Cryoglobulinemic membrano-proliferative glomerulonephritis**	Sjögren syndrome [2,6]
**Minimal change disease**	Sjögren syndrome [6,24], Polymyositis [52], Systemic lupus erythematosus [82,83], Antiphospholipid syndrome [124], Rheumatoid arthritis [17]
**Membranous nephropathy**	Sjögren syndrome [2,6], Polymyositis [53], Dermatomyositis [55-57], Class V lupus nephritis [78], Antiphospholipid syndrome [124], Rheumatoid arthritis [17,133-135]
**Secondary renal amyloidosis**	Sjögren syndrome [18], Rheumatoid arthritis [17,136,137]
**Thrombotic microangiopathy**	Antiphospholipid syndrome [119,123], Scleroderma renal crisis [13]
**Diffuse proliferative glomerulonephritis**	Dermatomyositis [49]
**IgM nephropathy**	Systemic lupus erythematosus [81]
**Collapsing glomerulopathy**	Systemic lupus erythematosus [79]
**Lupus nephritis**	Class I to VI lupus nephritis [78], drug-induced proliferative lupus nephritis [142]
**Fibrillary glomerulonephritis**	Antiphospholipid syndrome [125], Rheumatoid arthritis [140]
**Necrotizing crescentic glomerulonephritis (including drug-induced forms)**	Scleroderma [35], Rheumatoid arthritis [141]
**Focal proliferative glomerulonephritis**	Rheumatoid arthritis [17]
**Crescentic GN with FSGS**	Polymyositis [61]
**C3 nephropathy**	Antiphospholipid syndrome [124]

## Review

### Sjögren syndrome

#### Introduction

Primary Sjögren syndrome (PSS) is an autoimmune disorder of hitherto unknown origin which is characterized by polyclonal B-cell activation as well as lymphocytic infiltration of the exocrine glands, resulting in keratoconjunctivitis sicca (dry eyes disease) and/or xerostomia (dry mouth disease) [18]. In addition, extraglandular manifestations of PSS can affect organ systems, such as the lungs, blood vessels, skin, the gastrointestinal tract, central and peripheral nervous system, muscular skeletal apparatus and the kidney [19,20]. Affected patients are at increased risk of developing non-Hodgkin’s lymphoma, in particular lymphomas of B-cell origin [21]. Secondary Sjögren syndrome is associated with other rheumatic disorders, such as RA, SLE, SSc and others. Renal disease with PSS is reported to occur in 4.2% [1] to 67% [2] of patients. The variation is considered to be associated with the different diagnostic criteria used, different study designs and small cohorts examined as well as selection bias.

#### Histopathology/kidney involvement

Acute or chronic tubulointerstitial nephritis (TIN) with defects in tubular function is the predominant lesion in biopsy-proven renal involvement [2,6]. Distal (type I) renal tubular acidosis (RTA) is the most common clinical finding, leading to mild symptoms but also to potentially life-threatening complications, such as hypokalemic paralysis [22]. Albeit considered to be rare, proximal (type II) RTA has been reported in some cases [6,23]. Moreover, glomerular disease, such as cryoglobulinemic membrano-proliferative glomerulonephritis, focal segmental glomerulosclerosis (FSGS), mesangial proliferative glomerulonephritis, membranous nephropathy and minimal change disease have been reported [2,6,7,24]. A single case of long-lasting TIN in a patient with PSS led to a secondary (AA) amyloidosis with, consequently, renal failure and nephrotic syndrome [18]. Interestingly, in one study SSA/Ro, SSB/La and rheumatoid factor, as well as hypergammaglobulinemia, were detected in all subjects with biopsy-proven renal involvement [6]. In another cohort, all patients with distal RTA had positive anti-nuclear antibodies and either SSA or SSB antibodies were detected in 85.7% of them [25].

#### Therapy

Treatment with glucocorticoids should be initiated as first line therapy in patients with PSS and renal involvement since a good response to early treatment has been reported [6,26]. In addition, long-term bicarbonate and/or electrolyte supplementation should be commenced in a majority of patients to prevent life-threatening complications [26]. Besides corticosteroids, alternative immunosuppressive therapies (hydroxychloroquine, rituximab, cyclophosphamide) should be prescribed based on kidney biopsy findings as well as comorbidities. It was shown that renal function maintained or improved during a median follow-up period of 38 months after treatment with immunosuppressive drugs [6].

#### Conclusion

Corticosteroids are a mainstay in the treatment of TIN. Further histologic findings require specifically-tailored immunosuppression and most importantly, supplementation of bicarbonate and/or electrolytes, when indicated, should be commenced.

### Scleroderma renal crisis

#### Introduction

SSc is a CTD characterized by deposition and overproduction of extracellular matrix proteins and collagen, resulting in tissue fibrosis and, subsequently, tissue dysfunction. Affected organs and tissues include the skin, gastrointestinal tract, heart, lungs and kidneys. Involvement of the vascular system generally results in the development of Raynaud’s phenomenon early in the disease course. Consecutively, severe clinical manifestations of vascular dysfunction can be observed in some patients leading to pulmonary fibrosis and pulmonary artery hypertension, esophageal motility dysfunction, watermelon stomach, cardiac involvement, as well as scleroderma renal crisis (SRC) [27-29]. Epithelial to mesenchymal transition (EMT), a condition that conveys a phenotypic conversion from differentiated epithelial cells to matrix-producing fibroblasts and myofibroblasts, is recognized as a crucial part of the development of tissue fibrogenesis [30,31]. Several growth factors, such as transforming growth factor ß (TGFß) [8], connective tissue growth factor (CTGF) [9], as well as other mediators, such as endothelin-1 [10], are involved in tissue remodelling. SRC occurs in roughly 5% of patients with SSc [3]. Several risk factors with a predictive value were established: duration of SSc onset of less than four years, higher incidence of progressive skin thickening prior to renal involvement, new development of anemia and cardiac involvement (pericardial effusion or congestive heart failure) [32]. Detection of anti-RNA polymerase III antibodies displays a strong risk marker for the presence of SRC, whereas the presence of anti-topoisomerase and anti-centromere antibodies in scleroderma indicates a favorable disease course [33]. In addition, a case control study revealed a significant positive association between long-lasting high-dose corticosteroid treatment (≥15 g/d) and the onset of SRC [34]. On average, in 10% of patients, SRC occurs in the absence of hypertension. Normotensive renal crisis was more frequently present in patients treated with high doses of corticosteroids and in patients with redundant microangiopathic hemolytic anemia and thrombocytopenia in consequence to the underlying disease [11]. In addition, normotensive renal failure in SSc was associated with a higher mortality rate and an earlier need for dialysis treatment [11-13]. Blood pressure levels greater than 150/90 mmHg were observed in almost 90% of patients experiencing SRC. Hypertensive SRC is accompanied by clinical signs of malignant hypertension with left ventricular failure, hypertensive encephalopathy and arrhythmia [12].

#### Histopathology/kidney involvement

Diagnosis of SRC is confirmed by renal biopsy, which shows a thrombotic microangiopathic process, particularly affecting small vessels. Vascular changes are accompanied by thrombosis, accumulation of myxoid material and later in the disease course, development of onion-skin lesions and/or fibrointimal sclerosis [13]. Furthermore, one has to keep in mind that ANCA-associated vasculitis is a rare complication of SSc and in general presents with antibodies directed against myeloperoxidase and p-ANCA [35].

#### Therapy

Early use of ACE inhibitors, on the basis of most experience in particular captopril, is undoubtedly a cornerstone in the management of hypertensive SRC. Immediate use with a progressive increase of ACE inhibitor dosage, even in the presence of deteriorating kidney function, is considered to prevent or even reverse renal failure [36,37]. Additional antihypertensive therapy (calcium channel blockers, alpha/beta-adrenoreceptor antagonists and/or minoxidil) is mandatory when blood pressure is insufficiently controlled [28,36]. Recent findings suggest that dialysis was required in more than 50% of patients either in case of volume overload together with renal deterioration or to control blood pressure due to therapy-resistant hypertension [3,12]. Discontinuation of dialysis treatment could be accomplished in 16 to 55% patients with SRC [3,37]. After a stable disease course with continuous dialysis, renal transplantation should be considered when contraindications are ruled out. In a cohort of 260 patients with SSc who underwent kidney transplantation, the overall five-year graft survival rate was 56.7%. Among those, the recurrence of disease after transplantation was 6.7% in a report of the United Network of Organ Sharing (UNOS) [38]. Based on the finding that cyclosporine A (CSA) may be responsible for acute renal failure in patients with SSc [39], calcineurin inhibitors are not generally recommended as immunosuppressants after kidney transplantation.

#### Conclusion

Renal involvement in SSc is often accompanied by progressive renal failure and rapid initiation of therapeutic interventions is mandatory. Blood pressure control, in particular with ACE-inhibitors and additional antihypertensive medication, is essential. If blood pressure is not adjustable or the patient shows signs of fluid overload, dialysis should be considered early in the disease course. Discontinuation of dialysis was reported in some patients. In patients with chronic hemodialysis and renal transplantation, a calcineurin-inhibitor free immunosuppressive regimen might be chosen.

### Dermatomyositis and polymyositis

#### Introduction

Auto-immune myopathies, namely dermatomyositis (DM) and polymyositis (PM), share common clinical features, such as proximal muscle weakness, muscle inflammation, presence of autoantibodies, elevated muscle enzymes, electromyographic alterations and extra muscular manifestations. Despite clinical similarities, both differ regarding muscle biopsy findings and DM is associated with cutaneous involvement. The presence of a heliotrope rash, which is characterized by a violaceous skin discoloration around the eyes, and Gottron’s sign (erythematous papules with involvement of joints) are pathognomonic for DM [40,41]. Both entities are associated with concurrent incidence of neoplasms. In large cohorts, malignancies were detected in 9.4 to 32% of patients in DM and in 4.4 to 17% in PM patients [42-45] with a predominance of adenocarcinomas [45].

#### Histopathology/kidney involvement

Two types of renal involvement have been described in patients with PM/DM. First, rhabdomyolysis with release of myoglobin can lead to acute tubular necrosis with deterioration of renal function [14,15]. Second, several reports revealed the occurrence of chronic glomerulonephritis in patients with PM/DM [14,46-49]. In PM, mesangial proliferative glomerulonephritis represents the leading glomerular lesion [46,50,51]. Moreover, other biopsy specimens showed lipoid nephrosis with FSGS [52], membranous nephropathy [53] and crescentic glomerulonephritis with FSGS [54]. In contrast, the predominant finding in DM with renal involvement is membranous nephropathy [55-57]. Nevertheless, both mesangial proliferative glomerulonephritis [58] and diffuse proliferative glomerulonephritis [49] have been reported in single case reports.

#### Therapy

High-dose oral corticosteroids are the cornerstone of DM/PM therapy. Moreover, the addition of immunosuppressive drugs, such as azathioprine (AZA) or cyclophosphamide (CYC), as well as anti-malaria medication in DM and methotrexate, CYC, intravenous immunoglobulins and CSA in PM has been reported to improve the renal outcome in DM/PM [14,49,55-58]. In contrast to these reports, one patient progressed to end-stage renal disease despite immunosuppressive treatment [59]. Follow-up of the patients with DM revealed a high mortality rate due to cancer or multi-organ failure, while mortality in PM was high due to acute rhabdomyolysis followed by severe hyperkalemia and metabolic acidosis in a case report [14].

#### Conclusion

Management of patients with auto-immune myopathies and renal involvement require special caution, because disease-related mortality due to rhabdomyolysis and hyperkalemia is greatly feared. Special therapeutic intervention with immunosuppression should be tailored to the underlying histology. In most cases, corticosteroids might be effective as one therapeutic component.

### Systemic lupus erythematosus

#### Introduction

SLE depicts a remarkable complex autoimmune disease with considerable heterogeneity in clinical manifestations and disease course. Classification of SLE was last edited by the American College of Rheumatology (ACR) in 1997 [60] (Table 2). Earlier diagnosis, more intensive treatment regimens and diverse alternative strategies and possibilities to treat co-morbidities have contributed to improvement of prognosis [61]. Negative predictive factors with respect to survival include male gender, positive lupus anticoagulant, glomerulonephritis and “severe” onset of SLE [62]. The incidence is much higher in young woman and the prevalence is two- to four-fold greater in non-Caucasian populations [63]. Genetic, environmental and hormonal factors have been identified as possible risk factors for developing SLE [64,65].

**Table 2:** Revised criteria of the American College of Rheumatology


1. Malar rash	Fixed erythema, flat or raised, over the malar eminences, tending to spare the nasolabial folds
2. Discoid rash	Erythematous raised patches with adherent keratotic scaling and follicular plugging; atrophic scarring may occur in older lesions
3. Photosensitivity	Skin rash as a result of unusual reaction to sunlight, by patient history or physician observation
4. Oral ulcers	Oral or nasopharyngeal ulceration, usually painless, observed by physician
5. Non-erosive arthritis	Involving two or more peripheral joints, characterized by tenderness, swelling or effusion
6. Pleuritis/Pericarditis	1. Pleuritis, convincing history of pleuritic pain or rubbing heard by a physician or evidence of pleural effusion, or
	2. Pericarditis, documented by electrocardiogram or rub or evidence of pericardial effusion
7. Renal disorder	1. Persistent proteinuria >0.5 grams per day or more than 3+ on urine dipstick testing, or
	2. Cellular casts (may be red cell, hemoglobin, granular, tubular, or mixed)
8. Neurologic disorder	1. Seizures, in the absence of offending drugs or known metabolic derangements; for example, uremia, ketoacidosis, or electrolye imbalance, or
	2. Psychosis, in the absence of offending drugs or known metabolic derangements; for example, uremia, ketoacidosis, or electrolyte imbalance
9. Hematologic disorder	1. Hemolytic anemia with reticulocytosis, or
	2. Leukopenia <4.000/mm^3^ on ≥2 occasions, or
	3. Lymphopenia <1.500/mm^3^ on ≥2 occasions, or
	4. Thrombocytopenia <100.000/mm^3^ in the absence of offending drugs
10. Immunologic disorder	1. Anti-DNA: antibody to native DNA in abnormal titer, or
	2. Anti-Sm: presence to antibody of SM nuclear antigen, or
	3. Positive finding of antiphospholipid antibodies on:
	● An abnormal serum level of IgG or IgM anticardiolipin antibodies
	● A positive test result for lupus anticoagulant using a standard method, or
	● A false-positive test result for at least six months confirmed by *Treponema pallidum* immobilization or fluorescent treponemal antibody absorption test
11. Positive anti-nuclear antibody	An abnormal titer of anti- nuclear antibody by immunofluorescence or an equivalent assay at any point in time and in the absence of drugs

Diagnosis of systemic lupus erythematosus requires at least 4 out of 11 criteria [60]. Reprint permission was obtained from John Wiley and Sons, Inc.

Autoantibodies are directed against various nuclear antigens, in particular against chromatin components, such as nucleosomes, histones, anti-nuclear antibodies (ANA), double-stranded DNA antibodies (dsDNA) and ribonucleoproteins. Recently, it was suggested that the nucleosome might be the driving autoantigen in SLE. This hypothesis is supported by the finding that glomerular deposition of anti-dsDNA antibodies in lupus nephritis is mediated by nucleosomes [66,67]. The kidneys are a major source of autoantibody-producing plasma cells in lupus nephritis and these differentiated plasma cells are frequently observed in patients with severe renal involvement (mainly classes III through V), potentially acting in amplifying the renal disease course [68]. Additional autoantibodies include anti-Smith (Sm) antibodies with a high specificity for SLE, while SSA and SSB are present in other CTDs as well [69]. Complement levels are frequently reduced in patients with active disease. Genetic complete complement deficiencies can resemble a SLE-like disease [70]. Levels of complement C3 and C4 correlate with the overall disease activity. Patients with active lupus nephritis had significantly lower levels of C3 and C4 compared to patients with inactive lupus nephritis [71]. Serum C3 has generally higher sensitivity than serum C4, but both tests have only modest specificity for active lupus nephritis [72]. Assessment of the relationship between serum levels of C3 or C4 and renal flares revealed that C4 is critical for initiating a renal flare, while C3 activation is involved in the actual tissue damage [73]. Antibodies directed against C1q were detected in all patients with active nephritis in a large cohort [74]. Moreover, anti-C1q antibodies showed the strongest association with proteinuria among potential biomarkers and were significantly correlated with Renal Activity Score [75]. However, contradictory to these reports, in a cohort of 126 patients, anti-C1q antibodies were not significantly associated with active lupus nephritis [76].

#### Histopathology/kidney involvement

Involvement of the kidney in the natural history of disease is present in a majority of patients and is supposed to appear in almost 50% in the first year of diagnosis [4]. Recent findings even suggest a higher incidence, since a considerable proportion of patients with SLE have silent lupus nephritis. Diagnosis in the latter group was significantly earlier compared to the overt lupus nephritis group and urinary sediment as well as renal function tests were normal [77]. Renal biopsy findings are categorized according to the current classification of lupus nephritis, which was published on behalf of the International Society of Nephrology (ISN)/Renal Pathology Society (RPS) [78] (Table 3). However, one should be aware that other glomerular changes, such as collapsing glomerulopathy [79], IgA nephropathy [80], FSGS, IgM nephropathy [81], minimal change disease [82]/glomerular podocytopathy [83] can occur as well and alterations in kidney function due to rhabdomyolysis with acute kidney failure [84], as well as type I and IV RTA [85], have also been reported.

**Table 3:** **Revised classification lupus nephritis according to the International Society of Nephrology/Renal Pathology Society (ISN/RPS) 2003**[78]


**Class I**	**Minimal mesangial lupus nephritis**
	Normal glomeruli by light microscopy, but mesangial immune deposits by immunofluorescence
**Class II**	**Mesangial proliferative lupus nephritis**
	Purely mesangial hypercellularity of any degree or mesangial matrix expansion by light microscopy, with mesangial immune deposits
	May be a few isolated subepithelial deposits visible by immunofluorescence or electron microscopy, but not by light microscopy
**Class III**	**Focal lupus nephritis**
	Active or inactive focal, segmental or global endo- or extracapillary glomerulonephritis involving <50% of all glomeruli, typically with focal subendothelial immune deposits, with or without mesangial alterations
**Class III (A)**	Active lesions: focal proliferative lupus nephritis
**Class III (A/C)**	Active and chronic lesions: focal proliferative and sclerosing lupus nephritis
**Class III (C)**	Chronic inactive lesions with glomerular scars: focal sclerosing lupus nephritis
**Class IV**	**Diffuse lupus nephritis**
	Active or inactive diffuse, segmental or global endo- or extracapillary glomerulonephritis involving ≥50% of all glomeruli, typically with diffuse subendothelial immune deposits, with or without mesangial alterations. This class is divided into diffuse segmental (IV-S) lupus nephritis when ≥50% of the involved glomeruli have segmental lesions, and diffuse global (IV-G) lupus nephritis when ≥50% of the involved glomeruli have global lesions. Segmental is defined as a glomerular lesion that involves less than half of the glomerular tuft. This class includes cases with diffuse wire loop deposits but with little or no glomerular proliferation
**Class IV-S (A)**	Active lesions: diffuse segmental proliferative lupus nephritis
**Class IV-G (A)**	Active lesions: diffuse global proliferative lupus nephritis
**Class IV-S (A/C)**	Active and chronic lesions: diffuse segmental proliferative and sclerosing lupus nephritis
	Active and chronic lesions: diffuse global proliferative and sclerosing lupus nephritis
**Class IV-S (C)**	Chronic active lesions with scars: diffuse segmental sclerosing lupus nephritis
**Class IV-G (C)**	Chronic inactive lesions with scars: diffuse global sclerosing lupus nephritis
**Class V**	**Membranous lupus nephritis**
	Global or segmental subepithelial immune deposits or their morphologic sequelae by light microscopy and by immunofluorescence or electron microscopy, with our without mesangial alterations
	Class V lupus nephritis may occur in combination with class III or IV in which case both will be diagnosed
	Class V lupus nephritis show advanced sclerosis
**Class VI**	**Advanced sclerosis lupus nephritis**
	≥90% of glomeruli globally sclerosed without residual activity

Reprint permission was obtained from the Journal of the American Society of Nephrology.

#### Therapy

In general, use of ACE inhibitors significantly reduced the development of proteinuria and/or biopsy-proven lupus nephritis and was associated with a decreased risk of disease activity [86]. Concomitant use of antimalarial drugs (chloroquine and hydroxychloroquine) at diagnosis of lupus nephritis reduced the risk of progression to end-stage renal failure and frequency of hypertension [87].

Specific treatment follows the class of lupus nephritis, which is defined by the revised ISN criteria. Class I and class II require no therapy directed at the kidney in consequence of good long-term renal outcome [88]. In contrast, high-dose steroid therapy rapidly resolved nephrotic syndrome in a majority of SLE patients with minimal change disease either in the absence or with underlying class II lupus nephritis based on renal biopsy findings [82,83]. Immunosuppressive treatment is required in the management of class III (focal), class IV (diffuse) and class V (membranous nephropathy) lupus nephritis and usually consists of high dose glucocorticoid therapy along with intravenous CYC or mycophenolate mofetil (MMF) as induction therapy. The Euro Lupus Nephritis Trial compared low dose CYC (fortnightly, at a fixed dose of 500 mg, with a cumulative dose of 3 g) with the previously established high dose CYC (NIH) regimen (mean cumulative dose 8.5 g). Both strata were followed by AZA as remission-maintaining treatment. Renal outcome was similar in both treatment arms, but the low dose CYC group had fewer severe infections, even though the difference was not statistically significant [89]. Reports from this trial with a 10-year duration of follow-up confirmed the efficacy of the Euro Lupus regimen [90]. Since a majority of the subjects were white in this trial, these results might be less applicable to other ethnicities. In a more diversified cohort (>50% blacks) MMF (mean daily dosage 2.68 g) has been reported to be superior as induction therapy when compared to monthly CYC (0.5 g to 1 g/m^2^) in patients with class III through V lupus nephritis [91]. A further large, multi-center trial in a balanced cohort with respect to ethnicities designed to show superiority of MMF (mean daily dosage 2.47 g) to CYC failed to meet the primary end point. Both treatment arms achieved virtually identical rates of complete and partial remission. Furthermore, no significant difference with regard to severe adverse events or infections was reported [92]. Response to MMF as induction treatment in pure class V (membranous nephropathy) lupus nephritis in patients with diverse racial background appeared to show no difference in comparison to CYC [93]. Patients (>60% black) with class V lupus nephritis showed a better response regarding induction of remission after CSA when compared to CYC, whereas relapse of nephrotic syndrome occurred more frequently in patients with prior CSA therapy [94]. In a small cohort, multi-target therapy (MMF and tacrolimus) in patients with class IV and class V lupus nephritis revealed a higher rate of complete remission with a good tolerability when compared to intravenous CYC [95].

Following induction therapy, long-term immunosuppression is mandatory to avoid severe flares and to maintain stabilization of disease activity. Thus, immunosuppressants with a favorable safety profile and good efficacy are mandatory. MMF and AZA are deemed suitable and have shown efficacy in maintaining remission of lupus nephritis [96]. Equivalence of MMF and AZA was reported in the MAINTAIN Nephritis Trial, even though a trend towards fewer renal flares in the MMF group (19% vs. 25% in the AZA group) was reported [97]. More recently, in a larger trial, MMF was superior to AZA with respect to maintaining a renal response and preventing relapse in patients with lupus nephritis [98].

B-cell depleting therapy with anti-CD20 antibody rituximab (RTX) proved efficient in patients with active SLE including patients with lupus nephritis, who were nonresponsive to standard immunosuppressive therapy [99]. In proof of the efficacy of RTX treatment in moderately to severely active SLE and lupus nephritis, two large multi-center trials were conducted. The EXPLORER trial (moderate to severe active SLE) demonstrated no difference in primary/secondary end points between RTX and placebo. In a subgroup analysis a beneficial effect of RTX was observed in the African-American/Hispanic subgroup [100]. In patients with proliferative lupus nephritis and background immunosuppression (MMF) no difference was noted when RTX was added with regard to safety and efficacy (LUNAR trial) [101] even though opportunistic infections are reported to be rather common in SLE patients related to RTX treatment [102]. Enthusiasm was also dampened by reports on the development of progressive multifocal leukoencephalopathy in SLE patients following treatment with RTX [103].

Novel approaches with focus on targeted therapy have been developed and are currently being evaluated in clinical trials. Circulating B-lymphocyte stimulator (BLyS) is elevated in SLE, and titers correlate with increased disease activity and elevated dsDNA antibody concentrations [104]. Patients with serologically active SLE responded significantly better to belimumab, an antibody that binds to BLyS and inhibits its biological activity, plus standard of care (SOC) than to SOC alone [105]. The efficacy of belimumab was further corroborated in two large phase III trials, BLISS 52 [106] and BLISS 76 [107]. In both trials, belimumab met its primary efficacy end point and was consequently approved by the FDA in the treatment of SLE with the exception of severe active lupus nephritis or central nervous system lupus. Further investigations addressed to evaluate the role in active lupus nephritis are necessary.

Promising results have been obtained in a phase II trial for epratuzumab, a humanized anti-CD22 antibody [108,109]. Atacicept, a soluble receptor fusion protein, neutralizes the activity of BLyS and a proliferation-inducing ligand (APRIL) and their heterotrimers [110]. In a phase I trial, atacicept was well tolerated and demonstrated a dose-dependent reduction of immunoglobulin levels and mature/total B cell numbers [111]. However, in patients with active lupus nephritis, a phase II trial was terminated due to an increased number of infections [109]. Further trials assessing the efficacy and safety are currently ongoing.

In patients with highly active lupus nephritis with failure of conventional therapy, short-term as well as prolonged immuno-adsorption led to a significant reduction in proteinuria and to sustained remission rates [112]. Autologous stem cell transplantation achieved sustained clinical remissions in patients refractory to conventional immunosuppressive treatment, even though this clinical benefit was associated with increased mortality rates in most studies conducted so far [113]. Intravenous immunoglobulins have shown benefits in patients nonresponsive to other therapies and as a steroid-sparing agent [114].

#### Conclusion

The histopathologic classification of lupus nephritis still guides the therapy. In proliferative lupus nephritis (III and IV), CYC and MMF have shown almost identical therapeutic responses as induction therapy in large trials. CSA might be an alternative to these immunosuppressive agents in pure class V lupus nephritis. In patients not responding to initial treatment, multi-target therapy might be an effective alternative. MMF seems to be superior to AZA in maintaining remission. The role of RTX in the treatment of lupus nephritis has to be further elucidated, as well as the significance of novel therapeutic approaches in the therapy of lupus nephritis.

### Kidney disease in antiphospholipid syndrome

#### Introduction

Antiphospholipid syndrome (APS) is defined by the association of vascular thrombosis potentially affecting all segments of the vascular bed, complications during pregnancy (including unexplained consecutive spontaneous abortions, premature births because of severe preeclampsia, eclampsia or placental insufficiency or unexplained death before the 10^th^ week of gestation), and the presence of antiphospholipid antibodies (aPL), namely anticardiolipin antibodies (aCL) and lupus anticoagulant (LAC) [115]. The APS is classified as primary APS in the absence of associated autoimmune disease, whereas secondary APS is found alongside other autoimmune disorders [116].

#### Histopathology/kidney involvement

Renal manifestations in the context of APS may result from thrombosis occurring at any location in the renal vasculature. Renal artery stenosis (RAS) is a common complication of APS, leading to renovascular hypertension [117]. In a retrospective study, patients with APS, RAS and hypertension receiving oral anticoagulation with a target trough International Normalized Ratio (INR) >3.0 had better blood pressure control and renal function remained stable or improved, while in patients with an INR <3.0 renal function significantly deteriorated and blood pressure was poorly controlled [118]. Arterial hypertension is a well-documented complication of APS. In a series of patients with primary APS, a large proportion of patients presented with hypertension, which was attributed to biopsy-proven vascular nephropathy [119]. Kleinknecht *et al*. reported that all patients had severe hypertension and renal insufficiency in a small cohort of patients with secondary APS due to SLE [120]. Thrombosis of the renal vein and inferior vena cava usually presents with nephrotic-range proteinuria in primary and secondary APS [121], especially in those with circulating LAC [122]. APSN refers to kidney damage caused by intrarenal vascular damage and may be acute, in case of the presence of thrombotic microangiopathy, and/or chronic, in the case of arteriosclerosis, fibrous intimal hyperplasia and focal cortical atrophy [119,123]. Thrombotic microangiopathy is characterized by distinctive microscopic and ultrastructural changes and clinical presentation commonly includes hypertension, mild to nephrotic-range proteinuria and renal impairment [119,123]. Tektonidou *et al.* examined kidney biopsies obtained from patients with SLE with or without presence of aPL. APSN was detected in almost 40% with aPL, compared with only 4.3% of patients without aPL [16]. Fakhouri *et al*. examined 29 kidney biopsies of patients with APS [124]. In nine of these biopsies predominant pathological features distinct from ASPN were noted: membranous nephropathy (three cases), minimal-change disease/focal segmental glomerulosclerosis (three cases), mesangial c3 nephropathy (two cases), and pauci-immune crescentic glomerulonephritis (one case). Furthermore, a case of fibrillary glomerulonephritis in a patient with APS was published recently [125]. Interestingly, the presence of aPL in patients undergoing renal transplantation significantly increases the risk of renal vascular thrombosis and graft failure [126,127].

#### Therapy

Blood pressure control is the key intervention in the treatment of APS-related renal involvement. Adequate anticoagulation (if evidence of microthrombi is present) has shown encouraging results in small cohorts and may prevent progression to end-stage renal disease [128]. Evidence supporting immunosuppressive therapy in these patients is limited to case series [125,129] and is not routinely recommended in APS-related renal manifestations. Contrasting, patients with catastrophic APS, which is characterized by severe multiple organ dysfunction in consequence of diffuse small vessel ischemia and thromboses predominantly affecting the parenchymal organs, usually receive a combination therapy, including anticoagulation, steroids, intravenous immunoglobulins and plasmapheresis, but despite this aggressive approach mortality is still high [130].

#### Conclusion

Blood pressure control is mandatory in patients with APSN. The role of anticoagulation with a target through INR above 3.0 in patients with APSN and microthrombi to prevent kidney function deterioration has to be elucidated in further, larger studies.

### Rheumatoid arthritis

#### Introduction

RA is characterized by persistent synovial, systemic inflammation and autoantibodies (particularly to rheumatoid factor and citrullinated peptides). Genetic as well as environmental factors contribute to the risk of developing RA [131]. Renal involvement is relatively common in patients with RA.

#### Histopathology/kidney involvement

A study of renal biopsy specimens indicated that mesangial glomerulonephritis is the predominant histopathologic finding in RA, followed by amyloidosis, membranous nephropathy, focal proliferative glomerulonephritis, minimal-change nephropathy and acute interstitial nephritis [17]. Development of membranous nephropathy is related either to therapy with disease modifying antirheumatic drugs (DMARDs), in particular gold thiomalate, D-penicillamine and bucillamine [132], and anti-TNF alpha therapy, such as etanercept and adalimumab [133,134], or rarely occurs concomitant with RA [135]. Secondary AA amyloidosis was prevalent in 5.8% of patients with RA and was accompanied by a shortened life expectancy [136]. Deposition of amyloid in renal tissue correlated significantly with parameters of renal function [132], while a lack of amyloid deposition in the glomerulus may characterize subjects with stable renal function [137]. Mesangial glomerulonephritis is probably related to RA itself, since its occurrence was associated with higher titers of rheumatoid factor (RF) when compared with RA patients without nephropathy. Deposition of mesangial IgA correlated with the duration of RA and elevated serum IgA levels, whereas mesangial IgM deposition was correlated with serum levels of IgM class RF [138]. In addition, single reports reveal the presence of FSGS [139] and fibrillary glomerulonephritis [140] in RA patients. Anti-TNF alpha therapy can be causative for the development of necrotizing crescentic glomerulonephritis and proliferative lupus nephritis [141,142]. Besides the renal side effects of gold salts, D-penicillamine and bucillamine, CSA as another DMARD has a serious potential for renal toxicity, which is manifested primarily in a decline in creatinine clearance [143].

#### Therapy

Improvement of clinical and laboratory parameters was achieved in most cases after drug withdrawal and in case of necessary initiation of immunosuppression [133,134,142,143]. In patients with amyloid deposition, etanercept treatment reduced proteinuria as well as serum amyloid A. Furthermore, it entailed a decrease in serum creatinine in patients with creatinine values <2.0 mg/dl at the onset of amyloidosis [144].

#### Conclusion

Therapy related deterioration of kidney function has to be excluded in patients with RA. In addition, the persisting inflammation can lead to deposition of amyloid. Thus, adequate therapy to reduce disease activity may be effective in preventing this late-onset complication. Specific therapeutic interventions should be tailored to the underlying histologic kidney involvement.

## Conclusion and future directions

Renal involvement is frequently present in CTDs and has variable phenotypes. Since there is a steady increase of knowledge regarding the pathophysiology behind auto-immune disorders, more specific therapeutic approaches have been developed and are currently in clinical trials.

Acute or chronic TIN is the predominant kidney biopsy finding in Sjögren syndrome. Kidney function normalizes in most cases after corticosteroids are initiated [2,6]. In addition, several glomerular lesion patterns have been described in Sjögren syndrome.

Results from hematopoietic stem cell transplantation (HSCT) in SSc are promising. Current studies, namely the SCOT and ASTIS trials, have completed patient recruitment and the first results are expected soon [145]. The ASSIST trial clearly depicted the efficacy of HSCT in patients with scleroderma, since all 10 patients in the HSCT-group improved when compared to none in the CYC–treated cohort [146]. In addition, endothelin receptor antagonists in combination with dual blockade of the renin-angiotensin-aldosterone system (RAAS) significantly reduced proteinuria and stabilized the serum creatinine level after an initial increase in a patient with SRC [147]. Despite efficacy in patients with pulmonary arterial hypertension in SSc [148], trials with the aim to show benefits of endothelin receptor antagonists in SRC have yet to be conducted.

Diverse glomerular alterations and rhabdomyolysis have been reported in patients with auto-immune myopathies. Guided therapy with the aim to treat the underlying disease improves kidney function in most cases.

In SLE, new therapeutic approaches have gained attention. One of these novel agents is belimumab, an inhibitor of serum BLyS, which was recently approved by the FDA for treatment of SLE with the exception of active lupus nephritis and central nervous system involvement. A randomized, controlled trial with inclusion of active lupus nephritis is currently being designed. Furthermore, BLyS inhibition may also be effective in the treatment of PSS, since patients with Sjögren syndrome exhibit increased BLyS levels [149]. In patients with SLE, B-cell depleting therapy with RTX was effective in a larger cohort including patients with lupus nephritis [99], and efficacy was furthermore confirmed in a recent meta-analysis evaluating patients with refractory lupus nephritis [150]. However, RTX failed to show superiority in two large phase III trials with patients either presenting without renal involvement (EXPLORER) or with renal involvement (LUNAR) [100,101] even though a *post hoc* analysis of the EXPLORER trial indicated that RTX-treated patients achieved lower disease activity without a subsequent severe disease flare when compared to those treated with placebo [151]. Persistent B-cell presence was associated with no clinical response following RTX treatment [152]. In addition, physicians should be aware of severe infectious complications following RTX treatment in SLE patients [102,103]. Despite other strategies, such as immunoglobulin administration, immuno-adsorption and stem cell transplantation [112-114], RTX is nevertheless one alternative in refractory SLE [99].

APS-related renal manifestation potentially affects any segment of the vascular bed and is commonly accompanied by arterial hypertension. Blood pressure control is crucial, whereas the role and the target level of oral anticoagulation needs to be further elucidated. Chronic inflammation, as well as drug related adverse effects, is causative of kidney involvement in RA. Etanercept has shown encouraging results in reduction of serum amyloid A in amyloidosis and patients with a baseline serum creatinine below 2 mg/dl tended to show a benefit following TNF-alpha inhibition [144].

Based on studies in non-diabetic nephropathy, patients with renal involvement in CTDs should receive RAAS blocking agents once proteinuria is >1 g/day [149,150]. Renal function needs to be monitored as well as serum potassium levels and blood pressure. In chronic kidney disease in the pre-dialysis state the lowering of LDL-cholesterol safely reduced the risk of major atherosclerotic events [153]. Accelerated atherosclerosis is a common finding in patients with chronic inflammation and in CTDs in particular [154]. Thus, modification of the risk factors contributing to the evolution of cardiovascular disease is crucial in these patients. Moreover, adherence to therapeutic advice may be an underestimated problem, since a recent study indicated that only one-quarter of patients with SLE had an adherence rate ≥80% [155]. In addition, counselling against smoking should be mandatory in patients with SLE and RA [156].

In summary, renal manifestations of CTDs are frequent. Renal biopsy to ensure diagnosis is necessary in most patients presenting with deterioration of renal function, increase of proteinuria or signs of nephritic syndrome (summarized in Table 4). An interdisciplinary approach to optimize treatment is the aim for patients with CTDs.

**Table 4:** Suggested kidney biopsy indications in connective tissue diseases


Biopsy indication	rapid deterioration of renal function (exclude post renal and pre renal disorders first)
Biopsy indication	proteinuria >1 g/d (measured by collecting urine; collection over the course of a 24-hour period; to begin urine collection, the patient voids and discards the urine already in the bladder, afterwards urine for the next 24 hours has to be collected to ensure accurate results), if other causes of proteinuria are ruled out
	the EULAR/ERA-EDTA recommendations for the management of lupus nephritis suggest performing a renal biopsy if reproducible proteinuria >0.5 g/d is present (especially with glomerular hematuria and/or cellular cases) [72]
Biopsy indication	nephritic urine sediment (red blood cell casts) with deterioration of kidney function (estimated GFR <60 ml/min) if pre-existing impaired renal function is ruled out
Consider re-biopsy	increase in proteinuria/serum creatinine despite ongoing immunosuppressive therapy (exclude post-renal and pre-renal disorders first); consider a repeat kidney biopsy due to potential phenotype change (for example, lupus nephritis)
Biopsy indication	suspected interstitial nephritis, findings of white blood cell casts; leukocyturia (due to proton pump inhibitors, non-steroidal anti-rheumatic drugs, Sjögren syndrome, rheumatoid arthritis, and so on)
Biopsy indication	diagnostic approach in case of uncertainties, when kidney involvement is suspected, but absolute indications are not met

Renal biopsy suggestions differ between centers due to local preferences. General recommendations are difficult to define and we would consider higher levels of proteinuria (>1 g/d) compared to the EULAR/ERA-EDTA recommendations as biopsy indication for patients with lupus nephritis in our center.

## Abbreviations

AA: Amyloid A; ACE: Angiotensin-converting-enzyme; aCL: Anticardiolipin antibodies; ACR: American college of rheumatology; ANA: Anti-nuclear antibodies; aPL: Antiphospholipid antibodies; APRIL: A proliferation-inducing ligand; APS: Antiphospholipid syndrome; APSN: Antiphospholipid syndrome nephropathy; AZA: Azathioprine; BLyS: B-lymphocyte stimulator; CSA: Cyclosporine A; CTD: Connective tissue disease; CTGF: Connective tissue growth factor; CYC: Cyclophosphamide; DM: Dermatomyositis; DMARD: Disease modifying antirheumatic drug; dsDNA: Double-stranded DNA antibodies; EMT: Epithelial to mesenchymal transition; FDA: Food and drug administration; FSGS: Focal segmental glomerulosclerosis; HSCT: Hematopoietic stem cell transplantation; INR: International normalized ratio; ISN: International society of nephrology; LAC: Lupus anticoagulant; LDL: Low-density lipoprotein; MMF: Mycophenolate mofetil; PM: Polymyositis; PSS: Primary sjögren syndrome; RA: Rheumatoid arthritis; RAAS: Renin-angiotensin-aldosterone system; RAS: Renal artery stenosis; RF: Rheumatoid factor; RPS: Renal pathology society; RTA: Renal tubular acidosis; RTX: Rituximab; SLE: Systemic lupus erythematosus; Sm: Smith; SRC: Scleroderma renal crisis; SOC: Standard of care; SSc: Systemic scleroderma; TGFß: Transforming growth factor ß; TIN: Tubulointerstitial nephritis; TNF: Tumor-necrosis factor; UNOS: United network of organ sharing.

## Competing interests

The authors declare that they have no competing interests.

## Authors’ contributions

AK performed the literature search and wrote the manuscript. GM critically reviewed the manuscript. Both authors approved the final version of the manuscript.

## Pre-publication history

The pre-publication history for this paper can be accessed here:

http://www.biomedcentral.com/1741-7015/11/95/prepub
